# Inadequate fetal heart rate monitoring and poor use of partogram associated with intrapartum stillbirth: a case-referent study in Nepal

**DOI:** 10.1186/s12884-016-1034-5

**Published:** 2016-08-19

**Authors:** Ashish KC, Johan Wrammert, Robert B. Clark, Uwe Ewald, Mats Målqvist

**Affiliations:** 1 International Maternal and Child Health, Department of Women’s and Children’s Health, Uppsala University Hospital, Uppsala, Sweden; 2 United Nation’s Children’s Fund, Nepal Country Office, UN House, Pulchowk, Nepal; 3 Latter-day Saint Charities, Salt Lake City, UT USA

**Keywords:** Intrapartum stillbirth, Fetal heart rate monitoring, Partogram, Clinical adherence, Nepal

## Abstract

**Background:**

Newborns are at the greatest risk for dying during the intrapartum period, including labor and delivery, and the first day of life. Fetal heart rate monitoring (FHRM) and partogram use to track labor progress are evidence-based techniques that can help to identify maternal and fetal risk factors so that these can be addressed early. The objective of this study was to assess health worker adherence to protocols for FHRM and partogram use during the intrapartum period, and to assess the association between adherence and intrapartum stillbirth in a tertiary hospital of Nepal.

**Methods:**

A case-referent study was conducted over a 15-month period. Cases included all intrapartum stillbirths, while 20 % of women with live births were randomly selected on admission to make up the referent population. The frequency of FHRM and the use of partogram were measured and their association to intrapartum stillbirth was assessed using logistic regression analysis.

**Results:**

During the study period, 4,476 women with live births were enrolled as referents and 136 with intrapartum stillbirths as cases. FHRM every 30 min was only completed in one-fourth of the deliveries, and labor progress was monitored using a partogram in just over half. With decreasing frequency of FHRM, there was an increased risk of intrapartum stillbirth; FHRM at intervals of more than 30 min resulted in a four-fold risk increase for intrapartum stillbirth (aOR 4.17, 95 % CI 2.0–8.7), and the likelihood of intrapartum stillbirth increased seven times if FHRM was performed less than every hour or not at all (aOR 7.38, 95 % CI 3.5–15.4). Additionally, there was a three-fold increased risk of intrapartum stillbirth if the partogram was not used (aOR 3.31, 95 % CI 2.0–5.4).

**Conclusion:**

The adherence to FHRM and partogram use was inadequate for monitoring intrapartum progress in a tertiary hospital of Nepal. There was an increased risk of intrapartum stillbirth when fetal heart rate was inadequately monitored and when the progress of labor was not monitored using a partogram. Further exploration is required in order to determine and understand the barriers to adherence; and further, to develop tools, techniques and interventions to prevent intrapartum stillbirth.

**Clinical trial registration:**

ISRCTN97846009.

## Background

Analyses of the average daily mortality rate for babies demonstrate a substantial rise in mortality at the initiation of labor and delivery, and throughout the first day of life [1, 2]. Of the estimated 1.2-million intrapartum stillbirths that take place globally, more than half of them occur in South Asia [3–5]. The increased risk for the fetus during the intrapartum period is strongly associated with intrapartum complications [1]. The use of fetal heart rate monitoring (FHRM) and partogram are crucial for early screening and identification of existing complications, so that early decision-making choices for additional interventions can be made [6].

FHRM is a method that helps to identify the early signs of fetal hypoxia; which may help to prevent stillbirth, early neonatal death, and long-term physical or mental disability by allowing healthcare providers to intervene [6, 7]. The preferred method of FHRM in low-risk labor is intermittent auscultation [8, 9]. However, there is a paucity of evidence regarding the effect of varying auscultation frequencies on intrapartum outcomes [7, 9]. In Nepal, intermittent auscultation every 15 to 30 min during labor, as recommended by WHO guidelines, is the protocol adopted in the national medical standards and taught in training packages for doctors, nurses and auxiliary nurses [10, 11]. The level of adherence to these standards by health workers, as well as its association with intrapartum outcomes, has not been evaluated previously.

A partogram is an inexpensive and relatively simple-to-use tool, which provides a continuous pictorial overview of the progress of labor, including indicators of both maternal and fetal wellbeing. Partogram use is thus attractive in low-resource settings, and is also recommended for use within the Nepal national medical standards [12, 13]. The use of the partogram, developed by the WHO, in the management of labor, reduces prolonged labor and has the potential to improve fetal outcome [14, 15].

In 2012, Nepal had an estimated stillbirth rate of 22.4 per thousand births with a total of 13,000 stillbirths occurring annually; of the total number of stillbirths, 20 % occur during the intrapartum period [4, 16]. Assessment of the quality of intrapartum care in 12 district and regional hospitals of Nepal has shown that compliance to the use of the partogram is inadequate, only one-fifth of deliveries were monitored using a partogram [17].

We conducted this study in a tertiary hospital in Nepal to assess the birth attendant’s adherence to protocols for FHRM, using intermittent auscultation, and partogram use during the intrapartum period, as well as to assess the association between both adherence to FHRM and partogram use, and intrapartum stillbirth.

## Methods

### Study design

We used a case-referent design nested within a larger cohort study. All women delivering in the hospital made up the source population, from which 20 % were randomly selected as referents. The referent population was selected at the time of admission in the hospital using the lottery technique. All women having intrapartum stillbirth during the study period were included in the case population. Any antepartum stillbirth occurring in the referent populations was excluded from this study, while any intrapartum stillbirth occurring in the referent population was re-categorized into the case population. The sample size of the study was based on the larger prospective cohort study, which aimed to detect a 20 % reduction in perinatal mortality with the statistical power of 80 and level of significance at 5 %.

Ethical approval for this study was received from Nepal Health Research Council (reg. 37/2012) and Uppsala University Sweden (dnr. 2012/267) as part of larger cohort study evaluating the impact of a Helping Babies Breathe quality improvement cycle [18, 19]. Written consent was obtained from each of the mothers prior to data collection.

### Setting

We conducted this study at Paropakar Maternity and Women’s Hospital; a tertiary, government-funded hospital located in Kathmandu, Nepal. The hospital has about 22,000 deliveries per year with an intrapartum stillbirth rate of nine per thousand births and an estimated 198-intrapartum stillbirths occurring annually [20]. The hospital provides comprehensive maternal care services with obstetricians, medical doctors and nurse midwives. The routine clinical protocol for assessment of women coming for delivery at the admission unit includes ascertainment of gestational age and assessment of fetal heart sound, obstetric complication, and stage of labor. Based on these assessments, and the risk category assigned, the woman is transferred to one of three different delivery units for intrapartum care (Table 1). This study was conducted over a period of 15 months from July 2012 to September 2013.

**Table 1:** Human resources and set-up of each of the delivery units at the hospital

Delivery units	Type of Health workers	Number of HW	Number of delivery beds	Type of delivery service
Maternal and Newborn Service Center	Nurse midwives	11	8	Low-risk delivery
Labor Room	Obstetricians, medical doctors, nurse midwives	11	9	Low- and high-risk delivery
Operation room	Anesthesiologist, obstetricians, medical doctors, nurse midwives	11	1	Cesarean section

### Participants

For the purpose of this study, all intrapartum stillbirths that occurred after hospital admission, i.e. woman who were in labor and had fetal heart sound at admission, were included as cases. Women that had fetal death at admission, i.e. absence of fetal heart sound, were excluded. Similarly, antepartum stillbirths occurring prior to onset of labor, were also excluded. All women who were randomly selected to be referents and who had a live birth, either by vaginal delivery or cesarean section for maternal or fetal indication, were included as the referent population. Intrapartum stillbirths occurring among women in the referent population were re-categorized and included in the case population.

### Data collection and management

A surveillance system was set up by recruiting 12 surveillance officers to be stationed in the admission, delivery, and postnatal units. Any woman admitted to the hospital for delivery was marked in the surveillance registry. From this sampling frame, study participants were randomly selected using a lottery technique. Specifically, an opaque jar with 100 balls was kept in the admission unit, of which 80 were white and 20 were yellow. For each admission, a ball was drawn from the opaque jar; if a yellow ball was drawn, the woman was enrolled into the study as part of the referent population. If the woman was selected as part of the referent population, the woman was tracked from the point of admission until discharge to assess labor progress and birth outcomes.

The surveillance officers stationed in delivery room enrolled at the women who had intrapartum stillbirth at the delivery room and took consent at the delivery room. The surveillance officers tracked all women who had intrapartum stillbirths occurring in the hospital. From both the referent and case populations, information on parity, previous obstetric and medical history, care during the current pregnancy, obstetric and medical complication during pregnancy and intrapartum care was retrieved from clinical record forms. The surveillance team conducted interviews with referent and case population at the time of discharge using a questionnaire in order to assess the woman’s social, demographic and household information.

The research manager, after receiving the completed clinical record and interview forms from the surveillance officers, checked them for completeness. Additionally, 10 % of clinical record forms were checked with the primary data source to ensure data accuracy. Data entry officers reassessed the completeness of forms, recoded the open-ended response questions, and entered the data from these checked forms into a CS-Pro database. To prevent data loss, indexing of all collected forms was done. After data entry and cleaning in the CS-Pro database was completed, the data were then exported to SPSS 17 for data analysis.

#### Variables

##### Intrapartum stillbirth

Fetuses delivered with a gestational age of more than 22 weeks and/or a birth weight of 500 g or more, with a fetal heart sound upon admission, but who died during the intrapartum period and thus had an Apgar score of 0 at 1 and 5 min without signs of maceration.

##### Adherence to FHRM as per the standard protocol

Adherence was considered adequate when the fetal heart rate was monitored in an interval of half an hour or less throughout the labour period.

##### Non-adherence to FHRM as per the standard protocol

Adherence was considered inadequate when any FHRM was monitored in a frequency of more than half an hour or monitored infrequently or not at all during the labour; frequency of monitoring categorized as 31–60 min, more than 60 min, or not at all.

##### Adherence to partogram use

Adherence was considered adequate when the partogram was used, i.e. filled in for the progress of cervical dilation and descent of the head every half an hour, to assess the progress of labor.

The information on obstetric complications during labor was retrieved from the clinical medical journal of each woman.

##### Antepartum hemorrhage

Vaginal bleeding occurring during labor, but before delivery, which was not mucus plug. The vaginal bleeding occurred with or without abdominal pain.

##### Hypertensive disorder

Occurred when the maternal diastolic blood pressure was 90 mmHg or more for two consecutive readings.

##### Mal-presentation

When the fetus presented in any other position than the vertex presentation.

##### Prolonged labor

When the cervix was not dilated beyond 4 cm after eight hours of regular contractions or if cervical dilatation was to the right of the alert line on the partogram.

##### Multiple pregnancies

If the woman was pregnant with more than one fetus.

##### Prolapsed cord

When the umbilical cord was present in the birth canal below the fetal presenting part or the umbilical cord was visible at the vagina following the rupture of membranes.

Gestational age of the baby:

- *Preterm birth*: Babies born before 37 completed weeks of gestation, estimated by the date of the mother’s last menstrual period or based on clinical examination of the newborn.
- *Term birth*: Babies who were born at, or after, 37 completed weeks of gestation, estimated by the mother’s last menstrual period or based on clinical examination of the newborn.
- *Maternal education* was categorized as women who had 5 year or less than 5 years of education (primary education) and those who had six or more years of education (secondary education or higher);
- *Ethnicity*: The group within the social system of Nepal to which the women’s family belongs [21]. The ethnicity was categorized as most advantaged (Brahmin/Chettri); relatively advantaged Janajatis (Newar, Gurung and Thakali); relatively disadvantaged Janajatis; relatively disadvantaged non-Dalit; most disadvantaged (Dalit and Muslim);

To the greatest extent possible, efforts were made to reduce potential biases by comparing background and social characteristics of referent women having live births with women having intrapartum stillbirths, and adjusting the associated factors in a multivariate regression model. The surveillance team was provided 5-day training on consent taking, data collection from the clinical record and interview with women participating in the study. To ensure the quality assurance of data collection, the research manager continuously trained the surveillance officers so as to reduce the differential misclassification and information bias.

### Statistical analysis

Analysis of adherence and non-adherence to FHRM and partogram use was assessed by mode and place of delivery among the all referent women using a Pearson’s chi-square test.

The comparison of background and social characteristics of the women in the referent and case populations was also done using Pearson’s chi-square test for categorical variables, as well as Fisher’s exact test. Maternal age, maternal education, ethnicity, antenatal care attendance, parity, obstetric complication during labor, sex of baby, birth weight, gestational age of baby, and mode of delivery of the women in the referent and case populations were compared.

Background and social characteristic variables were categorized as follows prior to completing comparative analysis. Maternal age was categorized into 5-year age intervals; maternal education as below or above the primary school level; ethnicity as Brahmin/Chettri (hill or terai), relatively advantaged Janjatis (Newar, Gurung, Thakali), disadvantaged Janjatis, non-Dalit (terai), Dalit (hill or terai) or Muslim; antenatal care attendance as having at least one ANC visit compared to no visits; and parity as primiparous, multiparous (1–2) or multiparous (3 or more). Obstetric complications during labor included antepartum hemorrhage, hypertensive disorder, prolonged labor, mal-presentation, and cord prolapse; these were aggregated into one category, i.e. the presence of at least one obstetric complication, and compared to cases in which there were no complications. Birth weight was categorized into three groups as less than 1500, between 1500–2499, and greater than or equal to 2500 g; gestational age in weeks, based on last menstrual period, was also categorized into three groups as less than or equal to 32, 33–36 and 37 or more weeks; and finally, mode of delivery was categorized as normal vaginal, instrumental or cesarean section delivery.

Univariate logistic regression was completed in order to determine the likelihood for intrapartum stillbirth based on those background characteristics that showed a difference (*p* < 0.001) between the referent and case populations, in order to measure the association. Additionally, univariate logistic regression was also used to test the association between FHRM and partogram use and intrapartum stillbirth, with intrapartum stillbirth as the dependent variable. Finally, multivariate logistic regression analysis was completed to again determine the association between both FHRM and partogram use and intrapartum stillbirth, including possible confounders determined through the univariate logistic regression analyses. For all analyses, a difference was considered significant when *p* < 0.05.

To the greatest extent possible, missing data was minimized for primary and secondary outcomes; however, there were missing data for some background characteristics of mothers, therefore we used the multiple imputation method to deal with data missing at random [22].

## Results

**Fig. 1:**
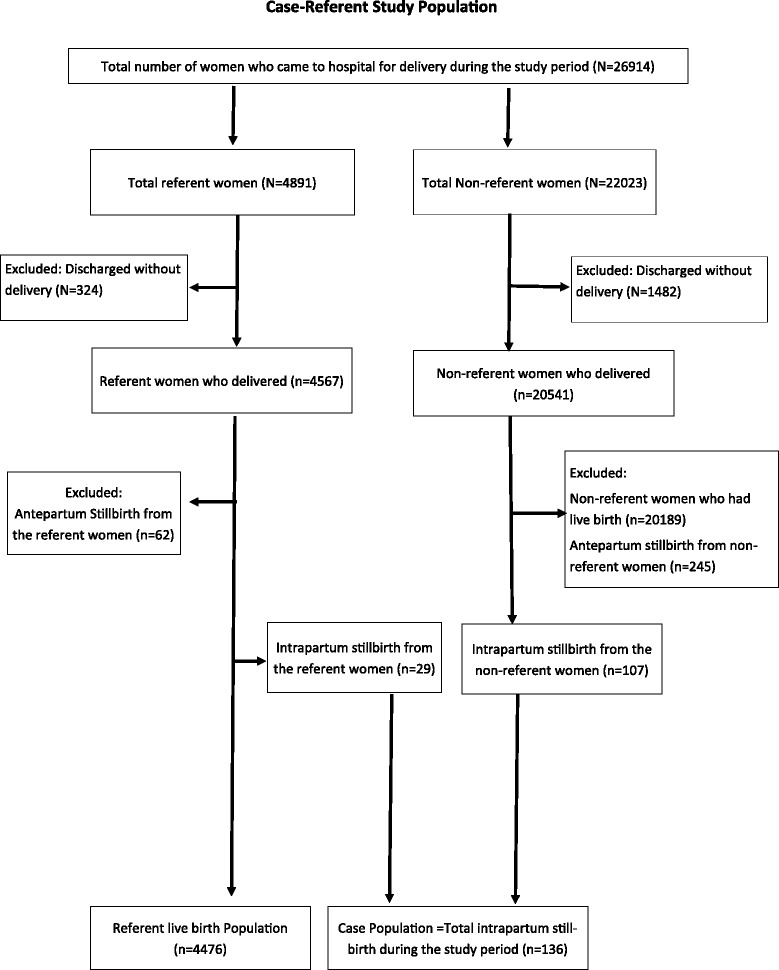
Flowchart of study population

During the study period a total of 26,914 women came to the hospital for delivery, of which 4,891 were selected as the referent population; however 324 mothers were discharged without delivering. Of the total referent population who delivered at the hospital, 4,476 infants were live-born and 91 were stillborn. Among the non-referent population there were 352 stillbirths. Thus, during the study period, 443 stillbirths occurred among the referent and non-referent populations combined, giving a stillbirth rate of 17.6 per thousand deliveries. Of the 443 stillbirths, 136 (30.7 %) were intrapartum stillbirths, giving an intrapartum stillbirth rate of 5.3 per thousand deliveries (Fig. 1).

**Fig. 2:**
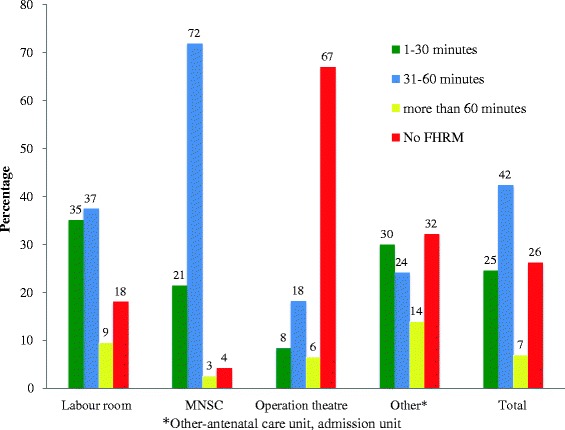
Frequency of FHRM based on place of delivery

Table 2 shows the frequency of FHRM use by mode of delivery. Only one-fourth of the women in labor were monitored as per the national standard for frequency of FHRM (every 1–30 min). About one-fourth of the women did not have any FHRM during the active stage of labor. One-half (49.3 %) of the women who delivered were monitored during the active stage at less-than-standard intervals (i.e. more than 30 min). Women who had a normal vaginal delivery were monitored more frequently than women who had an instrumental vaginal delivery or emergent cesarean delivery. More than one-third of the women in the labor unit had FHRM every 1to 30 min, compared to only 21 % in the lower-risk Maternal and Neonatal Service Center (MNSC) unit. However, in the labor unit, 18.4 % of deliveries did not receive any FHRM. Those deliveries taking place in the operation theatre, 67 % of them had no FHRM (Fig. 2).

**Table 2:** Frequency of intermittent fetal heart rate monitoring (FHRM) based on mode of delivery

Frequency of FHRM	Vaginal delivery	Instrumental delivery	Indicated Cesarean section delivery	Total
1–30 min	991 (29.5 %)	24 (22.4 %)	85 (8.4 %)	1100 (24.6 %)
31–60 min	1653 (49.2 %)	68 (63.8 %)	176 (17.4 %)	1897 (42.4 %)
>60 min	242 (7.2 %)	5 (4.7 %)	61 (6.0 %)	308 (6.9 %)
No FHRM	471 (14.0 %)	10 (9.3 %)	690 (68.2 %)	1171 (26.2 %)
Total	3357	107	1012	4476

**Fig. 3:**
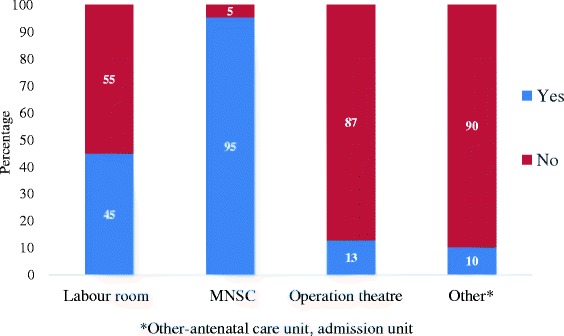
Utilization of partogram by place of delivery

Table 3 shows the relative use of the partogram to record the progress of labor by mode of delivery. Overall, intrapartum progress was recorded on the partogram for more than half of the women. The partogram was utilized in 68 % of the labors that resulted in instrumental vaginal delivery, but for only 12 % of the cesarean section patients. In the lower-risk MNSC unit, labor progress was recorded on the partogram for 95 % of women, whereas recording occurred for less than half of patients in the labor room and operation theater (Fig. 3).

**Table 3:** Utilization of partogram by mode of delivery

Mode of delivery	Partogram used		Total
	Yes	No	
Vaginal	2074 (61.8 %)	1283 (38.2 %)	3357
Instrumental	73 (68.2 %)	34 (31.8 %)	107
Indicated Cesarean section	125 (12.4 %)	887 (87.6 %)	1012
Total	2272 (58.8 %)	2204 (49.2 %)	4476

Table 4 shows the background and social characteristics of referent women with live births and women with intrapartum stillbirths. Analysis shows that there was a difference in the maternal age distribution among the referent and case populations (*p* < 0.001). Similarly there was difference in maternal education, antenatal care attendance, parity, occurrence of obstetric complications during labor, and infant birth weight and gestational age (*p* < 0.001).

**Table 4:** Maternal and child background characteristics of referent live births and intrapartum stillbirth cases

Variable	Referent Live Birth (*N* = 4476)	Intrapartum Stillbirth (*N* = 136)	*P*-value^a^
Maternal age in years			
Mean ± SD	23.7 ± 4.4	25.7 ± 6.3	
Median (IQR)	23.0 (20–26)	24.0 (20–30)	
	n (%)	n (%)	
Maternal age (5-year interval)			
<20	1224 (27.3)	34 (25.0)	
20–25	1957 (43.7)	45 (33.1)	
26–30	973 (21.7)	28 (20.6)	
>30	322 (7.2)	29 (21.3)	*p*<0.001
Maternal education			
Primary school (5 years) or less	1459 (32.6)	17 (12.5)	
Six years of schooling or more	3017 (67.4)	119 (87.5)	*p*<0.001
Ethnicity			
Brahmin/Chhetri (hill or terai)	1733 (38.7)	42 (30.9)	
Relatively advantaged Janajatis	812 (18.1)	22 (16.2)	
Disadvantaged Janajatis	1293 (28.9)	48 (35.3)	
Non-Dalit (terai)	369 (8.2)	12 (8.8)	
Dalit (hill and terai)	235 (5.3)	11 (8.1)	
Muslim	34 (0.8)	1 (0.7)	*p*=0.278
Antenatal Care Attendance			
At least one visit	3904 (87.2)	79 (58.1)	
No ANC	572 (12.8)	57 (41.9)	*p*<0.001
Parity			
Primipara	2418 (54.0)	64 (47.1)	
Multipara (1–2)	1869 (41.8)	51 (37.5)	
Multipara (3 or more)	189 (4.2)	21 (15.4)	*p*<0.001
Obstetric complication during labor*			
No	3965 (88.6)	69 (50.7)	
Yes	511 (11.4)	67 (49.3)	*p*<0.001
Sex of newborn			
Female	2103 (47.0)	52 (38.2)	
Male	2373 (53.0)	84 (61.8)	*p*=0.45
Birth Weight in grams			
VLBW (<1500)	40 (0.9)	37 (27.2)	
LBW (1500–2499)	474 (10.6)	42 (30.9)	
Normal BW (<2500)	3962 (88.5)	57 (41.9)	*p*<0.001
Gestational age in weeks			
<32	68 (1.5)	39 (28.7)	
33–36	295 (6.6)	23 (16.9)	
>37	4113 (91.9)	74 (54.4)	*p*<0.001
Mode of delivery			
Normal Vaginal delivery	3357 (75.0)	89 (65.4)	
Instrumental delivery	107 (2.4)	3 (2.2)	
Cesarean section delivery	1012 (22.6)	44 (32.4)	*p*=0.29

*Obstetric complications during labor included: antepartum hemorrhage, hypertensive disorder, mal-presentation, prolonged labor, and cord prolapse

^a^ p-value determined by Pearson’s chi-square test or Fisher’s exact test

Table 5 shows the univariate logistic regression analysis performed among the referent and case populations to determine associations with intrapartum stillbirth. The risk of intrapartum stillbirth increased with advancing maternal age, by 9 % for every year. The risk for intrapartum stillbirth was also positively associated with parity, with a 70 % increased risk for stillbirth with every additional previous delivery. Furthermore, there was an almost nine-fold increased risk for intrapartum stillbirth if the babies were born before 37 weeks of gestation, as compared to after. Similarly, stillbirth was almost 10 times more likely among low birth weight infants. Increased risk for intrapartum stillbirth was also associated with the lack of any antenatal care, the presence of any maternal complication, and indicated cesarean delivery. No significant associations with instrumental delivery or sex of the baby were detected. From these findings of univariate regression analysis, maternal age, parity, antenatal care attendance, obstetric complication during labor, and indicated cesarean section were added to the multivariate logistic regression model to test the association between the use of both FHRM and partogram, and intrapartum stillbirth.

**Table 5:** Association between intrapartum stillbirth and selected background characteristics of women and infants

Variables*	Crude odds ratio^a^	95 % CI
Maternal age in years	1.1	1.06–1.1
Parity	1.7	1.5–1.9
Gestational age in weeks		
37+0 or more	Ref	
36+7 or less	8.6	6.0–12.2
Birth weight in grams		
2500 or more	Ref	
Less than 2500	9.8	6.9–13.9
Antenatal Care Attendance		
Yes	Ref	
No	4.9	3.4–6.9
Obstetric Complication during intrapartum period		
No	Ref	
Yes	7.3	5.1–10.3
Mode of Delivery		
Vaginal delivery (normal or instrumental)	Ref	
Emergency C-section	1.7	1.1–2.4

* Variables selected based on significant differences (p<0.001) shown between the case and referent populations by Pearson’s chi-square test or Fisher’s exact test

^a^ Crude odds ratio determined through univariate logistic regression analysis for likelihood of intrapartum stillbirth

Table 6 shows the association between the use of intermittent FHRM and use of partogram with intrapartum stillbirth. FHRM not performed according to existing guidelines, i.e. less frequently than once every 30 min or no monitoring at all, showed significant association with intrapartum stillbirth. There was a four times higher risk of intrapartum stillbirth if FHRM was not performed according to guidelines (adjusted odds ratio, aOR, 4.17, 95 % CI 2.0–8.7), and an increasing risk with less frequent use, resulting in a seven-fold increased risk of intrapartum stillbirth if FHRM was performed less than every hour or not at all (aOR 7.38, 95 % CI 3.5–15.4). There was more than a three-fold increased risk for intrapartum stillbirth if the partogram was not used during the last stage of labor (aOR 3.31, 95 % CI 2.0–5.4).

**Table 6:** Association between intrapartum stillbirth and exposure variables (FHRM and partogram use)

	Live Birth (*n*=4476)	Intrapartum stillbirth (*n*=136)	Crude Odds Ratio^a^	95 % CI	Adjusted Odds Ratio^b^	95 % CI
Frequency of Fetal Heart Rate monitoring (FHRM)						
1–30 min	1100	9	Ref		Ref	
>30 min or no FHRM	3376	127	4.6	2.3–9.1	4.2	2.0–8.7
>60 min or no FHRM	1479	113	9.3	4.7–18.5	7.4	3.5–15.4
Use of Partogram						
Yes	2456	24	Ref		Ref	
No	2020	112	5.5	3.5–8.6	3.3	2.0–5.4

^a^Univariate logistic regression analysis to determine likelihood of intrapartum stillbirth

^**b**^Multiple logistic regression analysis to determine likelihood of intrapartum stillbirth based on FHRM frequency and partogram use, adjusted for maternal age (continuous), parity (continuous), low birth weight (<2500grms), prematurity (<37 weeks), ANC attendance, indicated Cesarean section, and presence of maternal risk factor

Analysis of the data restricting women with gestational age ≥37 weeks and ≥33 weeks separately showed that there were association of inadequate adherence of FHRM (≥60 min or none) and no use of partogram with intrapartum stillbirth after adjusting with the background variables which were significantly different in referent and case population.

## Discussion

Our study demonstrates that the adherence to FHRM every 30 min and use of partogram during the intrapartum period is inadequate in a tertiary-level hospital of Nepal. We found that there was an increased risk associated with intrapartum stillbirth when fetal heart rate was inadequately monitored and when progress of labor was not monitored using the partogram.

There were, however, some limitations in this study. First, this was a case-referent study and therefore could only show an association between the inadequate adherence to standard protocols and intrapartum stillbirth, but not a cause-and-effect relationship between them. Second, there might have been some potential biases, such as failure of the health workers to detect obstetric complications during labor or failure to detect the fetal heart sound, which might have led to a misclassification as an intrapartum stillbirth versus a neonatal death. Third, there may have been information bias, where the health worker failed to record FHRM or use the partogram even if measurements were done, which would be misclassified as FHRM or partogram not completed. Fourth, timing of enrolment of the case and referent was different, as the referent were enrolled at the time of admission while the case population were enrolled at the time of delivery. Fifth, there may have been data selectivity by the surveillance officers, either sub-consciously or consciously, between the case and referent populations as the outcome was known. Finally, we did not assess one of the potential confounder length of time from admission to delivery, so we could not adjust it in the analysis.

Inadequate adherence to standard clinical protocols has been a key challenge that has prevented the translation of evidence into good clinical practice, and subsequent improved clinical outcomes, especially in low- and middle-income settings [23, 24]. Overcoming these barriers by identifying context-specific solutions is key in reducing preventable intrapartum-related deaths.

One of our previous studies in the same hospital demonstrated that there was poor health worker performance on neonatal resuscitation, increasing babies’ risk for morbidity and/or mortality [25]. This poor performance has been identified as a key factor for the high number of intrapartum-related deaths.

The inadequate adherence to standard protocols for intrapartum monitoring can be due to a multitude of factors. These factors could range from lack of health institution leadership and/or support to improve clinical practice, shortages of staff, poor knowledge on the use of the partogram or FHRM, heavy workload for the inadequate number of staff, or lack of understanding of the relevance of the partogram in preventing obstructed labor as shown by studies in Africa [26–29]. Further exploration on possible contextual barriers to adherence to standard practices, and to identify possible interventions that help to facilitate adherence to standard protocols, is necessary. The potential reason for poor adherence to FHMR and use of partogram for caesarean deliveries in compared with vaginal delivery might be FHMR and partogram was not done after the decision was made for caesarean delivery.

## Conclusions

Improving the quality of intrapartum care in hospitals is essential in a context like Nepal, where there is an increase in health facility delivery with a limited number of human resources. To ensure that quality standard care is available during the intrapartum period, the period with the highest risk for adverse events in babies, more investment is required in order to determine and understand the barriers to adherence; and further, to develop tools, techniques and interventions to prevent intrapartum stillbirth.

## Abbreviations

aOR: Adjusted odds ratio
FHRM: Fetal heart rate monitoring
MNSC: Maternal and newborn service center
